# The Blood-Brain Barrier in Both Humans and Rats: A Perspective From 3D Imaging

**DOI:** 10.1155/2024/4482931

**Published:** 2024-08-26

**Authors:** Aiwen Chen, Gavin Volpato, Alice Pong, Emma Schofield, Jun Huang, Zizhao Qiu, George Paxinos, Huazheng Liang

**Affiliations:** ^1^ Shanghai Key Laboratory of Anesthesiology and Brain Functional Modulation, Shanghai, China; ^2^ Clinical Research Center for Anesthesiology and Perioperative Medicine Shanghai Fourth People's Hospital School of Medicine Tongji University, Shanghai, China; ^3^ Translational Research Institute of Brain and Brain-Like Intelligence Shanghai Fourth People's Hospital School of Medicine Tongji University, Shanghai, China; ^4^ Department of Anesthesiology and Perioperative Medicine Shanghai Fourth People's Hospital School of Medicine Tongji University, Shanghai, China; ^5^ Department of Acupuncture Shuguang Hospital Shanghai University of Traditional Chinese Medicine, Shanghai, China; ^6^ Department of Brain Structure and Function Neuroscience Research Australia, Randwick, New South Wales, Australia; ^7^ School of Medical Sciences The University of New South Wales, Kensington, New South Wales, Australia; ^8^ School of Chemical and Biomolecular Engineering The University of Sydney, Camperdown, New South Wales, Australia; ^9^ Centre of Life Science Suzhou Industrial Park Monash Research Institute of Science and Technology Southeast University-Monash University Joint Graduate School Monash University-Southeast University Joint Research Institute, Suzhou, Jiangsu Province, China

**Keywords:** 3D imaging, astrocyte, basal membrane, blood-brain barrier, endothelium, microglia, tissue clearing

## Abstract

**Background:** The blood-brain barrier (BBB) is part of the neurovascular unit (NVU) which plays a key role in maintaining homeostasis. However, its 3D structure is hardly known. The present study is aimed at imaging the BBB using tissue clearing and 3D imaging techniques in both human brain tissue and rat brain tissue.

**Methods:** Both human and rat brain tissue were cleared using the CUBIC technique and imaged with either a confocal or two-photon microscope. Image stacks were reconstructed using Imaris.

**Results:** Double staining with various antibodies targeting endothelial cells, basal membrane, pericytes of blood vessels, microglial cells, and the spatial relationship between astrocytes and blood vessels showed that endothelial cells do not evenly express CD31 and Glut1 transporter in the human brain. Astrocytes covered only a small portion of the vessels as shown by the overlap between GFAP-positive astrocytes and Collagen IV/CD31-positive endothelial cells as well as between GFAP-positive astrocytes and CD146-positive pericytes, leaving a big gap between their end feet. A similar structure was observed in the rat brain.

**Conclusions:** The present study demonstrated the 3D structure of both the human and rat BBB, which is discrepant from the 2D one. Tissue clearing and 3D imaging are promising techniques to answer more questions about the real structure of biological specimens.

## 1. Introduction

The neurovascular unit (NVU) is the fundamental combination of brain cells that determine the functions of the central nervous system [1, 2]. It is basically an expansion of the blood-brain barrier (BBB) to include surrounding neural cells which are interrelated functionally and anatomically [3, 4]. Under physiological conditions, the whole unit works collaboratively in regulating the blood flow to the brain region which is actively engaged in physiological functions, clearing or reuptaking neural transmitters, cleansing cellular or metabolic debris within the unit, and maintaining its homeostasis [5–7]. In diseased conditions, such as ischemic stroke, the microcirculation was injured due to ischemia, and the permeability of the BBB was increased [8]. Detrimental effects of blood contents and transgressed blood cells will aggravate the ischemic injury to the central nervous system [9]. Restoration of the BBB structure might be an effective method to minimize ischemic injury, at least secondary injury. To achieve this, the first step is to depict the entire structure of the BBB and how stimuli elicit injury to the BBB. Currently, it is known that the BBB is composed of endothelial cells, the basal membrane, pericytes, and astrocyte end feet surrounding the blood vessels [3, 4]. Literatures have described a typical diagram of the BBB, showing astrocyte end feet surrounding endothelial cells or the basal membrane or pericytes [10, 11]. But how these cells interact with each other in 3D has not been reported. Is every endothelial cell covered by pericytes or astrocytes? What is the percentage of the area of each endothelial cell covered by pericytes or astrocytes? These questions can not be answered by 2D images of the BBB.

With advances in tissue clearing and confocal microscopy or light sheet microscopy [12–16], it is possible to undertake fluorescent labelling in cleared tissues and obtain high-resolution microscopic images in 3D. A commonly used clearing technique—clear, unobstructed brain imaging cocktails and computational analysis, or CUBIC [13]—will enable us to examine the spatial relationship of these cells in 3D. In an attempt to investigate the difference in BBB structure between Alzheimer's disease patients and age-matched controls, we cleared thick slabs of human brain tissues obtained from the Sydney Brain Bank and imaged the endothelial cells, astrocytes, and microglial cells in 3D after immunofluorescence staining. In this study, we tentatively presented six videos showing the blood vessel endothelial cells and the basal membrane of blood vessels, microglial cells, and the spatial relationship between astrocytes and blood vessels in age-matched controls. Additional videos were obtained from the rat brain, which served as a comparison to human astrocytes and blood vessels.

## 2. Materials and Methods

### 2.1. Human Brain Tissue Preparation

Blocks of formalin-fixed postmortem human brain tissue were obtained from the middle temporal cortex of age-matched patients without brain lesions. Tissues were received from the Sydney Brain Bank, which is supported by Neuroscience Research Australia and the University of New South Wales. The experiments were approved by the Human Research Ethics Committee of the University of New South Wales (approval number: HC15751). Brain slices were manually cut out of blocks of the middle temporal cortex, spaced approximately 1 mm apart by using a 27-gauge surgical scalpel. Each resultant piece measured approximately 1 mm × 3 mm × the length of the cortex. As far as possible, each slice was taken perpendicular to the pial surface and included a section of white matter in order to ensure that the full length of the cortex was contained. These slices were then washed in PBS for > 24 h to wash out residual formalin, before undergoing the clearing procedure.

### 2.2. Rat Brain Tissue Preparation

Rats of 350 g were anesthetized with ketamine (80 mg/kg) and xylazine (5 mg/kg) diluted in 0.9% normal saline and perfused with normal saline first, followed by 4% paraformaldehyde solution in 0.01 M phosphate buffer. The brain tissue was then dissected from the skull and cut into thick sections (2 mm thick). The experimental procedure was approved by the Animal Care and Ethics Committee of the University of New South Wales (approval number: 14/94A).

### 2.3. Tissue Clearing

The CUBIC protocol used to clear human and rat brain tissue followed that described by Liang et al. [17], which is a variation of the CUBIC technique published by Susaki et al. [13]. In brief, CUBIC 1 clearing solution was prepared by dissolving 3.85 g of urea in 5.38 mL of warm distilled water, followed by adding 3.85 g of N,N,N',N'-tetrakis (2-hydroxypropyl) ethylenediamine to the solution. When the solution cooled down to room temperature, 2.31 g of Triton X-100 was added until the solution was homogeneous. Transfer the brain tissues to the solution in a 15 mL tube and keep the tube gently shaken on a rotating platform at 37°C until the tissues were transparent. Remove the CUBIC 1 solution and wash the tissues with PBS every 3 h for 12 h. Wash the tissues with 20% sucrose in PBS for 4 h at 37°C and then freeze the tissues in the mounting medium optimal cutting temperature (OCT) compound in a 15 mL tube overnight in a −80°C freezer. The rat brain tissue used for PDGFR*β*1 staining was cleared with a tissue-clearing device from Jiangsu Aoming Biotechnology Ltd.

### 2.4. Tissue Staining

The next day, the tissues were thawed and washed with PBS before incubating them in the primary antibody solutions (rabbit anti-Collagen IV, PA5-95188, Thermo Fisher, 1 : 100; goat anti-Glut1, sc-1605, Santa Cruz Biotechnology, 1 : 50; rabbit anti-Iba1, 019-19741, Wako, 1 : 200; goat anti-GFAP, SAB2500462, Sigma-Aldrich, 1 : 100; mouse anti-CD31, sc-71873, Santa Cruz Biotechnology, 1 : 100; rat anti-CD146, 134714, BioLegend, 1 : 100), respectively, for 7 days at 37°C. After four washes with PBS in a day, the tissues were incubated in the secondary antibody solutions (Alexa fluor 488 conjugated donkey anti-rabbit IgG, 711-545-152, 1 : 100; Alexa fluor 647 conjugated donkey anti-goat IgG, 705-605-003, 1 : 100; Alexa fluor 647 conjugated donkey anti-rabbit IgG, 711-605-152, 1 : 100; Alexa fluor 594 conjugated donkey anti-mouse IgG, 715-585-150, 1 : 100; Alexa Fluor 594 conjugated donkey anti-rat IgG, 712-585-150, 1 : 100, all from Jackson Immunoresearch Inc), respectively, for 7 days at 37°C. The tissues were then washed four times, every 3 h at 37°C.

### 2.5. Second Tissue Clearing and Imaging

Tissues were transferred to freshly prepared CUBIC 2 clearing solution which contained 50% (w/v) sucrose, 25% (w/v) urea, 10% (w/v) 2,2′,2^″^-nitrilotriethanol, and 0.1% (v/v) Triton X-100 and kept on a shaker in a 37°C oven until the tissues were transparent again. Load the tissues on a glass coverslip (24 × 60 mm) where two blue tack strips were placed on two sides. CUBIC 2 solution was dropped near the tissues, and the other glass coverslip was placed on the top of the blue tack strips. Press the top glass coverslip until it reach the brain tissue, and the CUBIC 2 solution was surrounding the tissues. Place them under either a confocal or two-photon microscope and scan the tissues at a step size of 2 *μ*m. Acquired images were saved as Leica Image File Format (.LIF) files, downsized from 1024 × 1024 to 512 × 512, and reordered to match the grid layout of the stitching software. These files were then opened with XuvStitch, the approximate alignment of tiles was configured, and automatic fine alignment and stitching were performed. The stitched images were saved as Bitplane: Imaris 5.5 (.IMS) files, opened with Bitplane Imaris, and cropped so that the white matter was removed and only the cortex—our region of interest—remained. Videos were then produced using these stacks of images in Imaris.

## 3. Results

Among the antibodies tested for imaging the BBB, all worked except CD13 and PDGFR*β*1 which are markers for pericytes (Table 1). Therefore, only images obtained from other antibody staining were presented.

**Figure 1:**
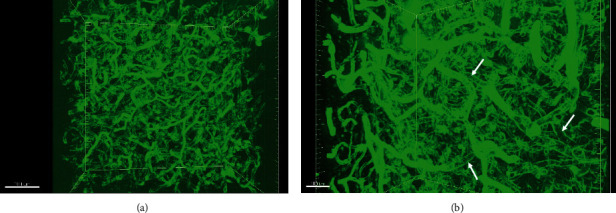
(a, b) Immunofluorescence staining against Collagen IV to show blood vessels in the human brain. Note the area with little collagen-positive signal (arrows). The scale bar is 200 *μ*m in (a) and 100 *μ*m in (b).

Collagen IV is a component of the basal membrane of the endothelial cells. Immunofluorescence staining against Collagen IV showed the entire profile of blood vessels in the human brain, ranging from capillaries to arterioles. Interestingly, the Collagen IV positive signal was not evenly distributed throughout the blood vessels with segments of certain blood vessels containing weak signals (arrows) (Figures 1(a) and 1(b)).

**Figure 2:**
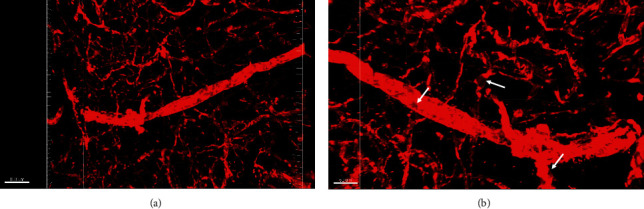
(a, b) Immunofluorescence staining against Glut1 to show blood vessels in the human brain. Note the patches with little Glut1-positive signal (arrows). The scale bar is 100 *μ*m in (a) and 50 *μ*m in (b).

Glut1 is a glucose transporter which is primarily present on the membrane of endothelial cells. Immunofluorescence staining against Glut1 showed positive signals not only in capillaries but also in larger vessels (Figures 2(a) and 2(b)). Similar to Collagen IV, Glut1 was also not evenly localized on the membrane of endothelial cells. Patches of positive signals were often present in small vessels like capillaries (arrows).

**Figure 3:**
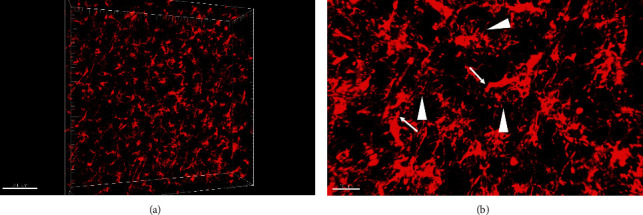
(a, b) Immunofluorescence staining against Iba1 to show microglial cells in the human brain. Note the thick branches (arrows) and thin processes (arrowheads). The scale bar is 200 *μ*m in (a) and 50 *μ*m in (b).

Iba1 is a marker for microglia. In the human brain, microglial cells and their short branches were clearly seen after immunofluorescence staining. The intensity of the positive signal was especially strong in the cytoplasm, and it was nearly impossible to see the empty nucleus. Both spindle-shaped and round-shaped microglial cells were stained. Processes derived from those branches were much thinner (arrowheads) than the branches, and they only extended to short distance (arrows) (Figure 3(a) and 3(b)).

**Figure 4:**
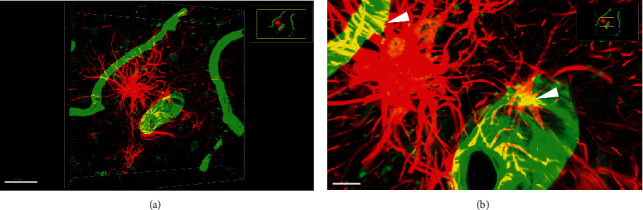
(a, b) Immunofluorescence staining against CD31 (endothelial cells) and GFAP (astrocytes) to show their spatial relationship in the human brain. Note the processes (arrowheads) of astrocytes wrapping blood vessels (arrows) and their overlap (arrowheads). The scale bar is 200 *μm* in (a) and 50 *μ*m in (b).

**Figure 5:**
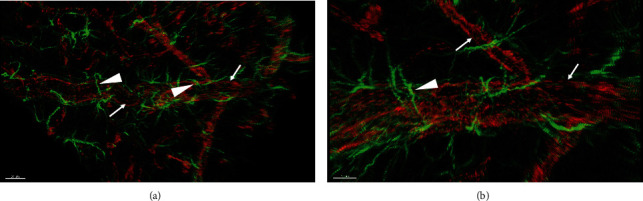
(a, b) Immunofluorescence staining against CD31 (endothelial cells) and GFAP (astrocytes) to show their spatial relationship in the rat brain. Note the processes (arrowheads) of astrocytes wrapping blood vessels and their overlap. Patches of blood vessels without CD31-positive signal were observed (arrows). The scale bar is 20 *μ*m in (a) and 10 *μ*m in (b).

**Figure 6:**
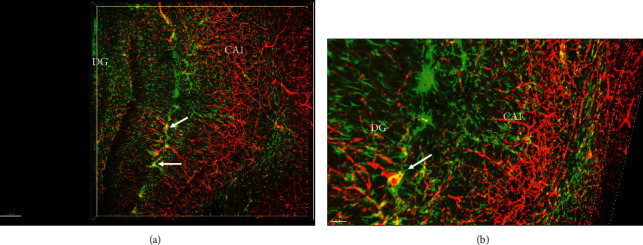
(a, b) Immunofluorescence staining against CD146 (pericytes) and GFAP (astrocytes) to show their spatial relationship in the rat hippocampus. Note the overlap between processes of astrocytes and pericytes (arrows). The scale bar is 150 *μ*m in (a) and 50 *μ*m in (b).

GFAP is a marker for astrocytes. In the human brain, GFAP-positive astrocytes were in close apposition with blood vessels (arrows) with numerous processes surrounding the blood vessels (arrowheads). However, their cell bodies were not that easily defined due to the presence of similar-sized processes around the cell body. It can be seen that the astrocyte processes did not wrap the entire area of blood vessels, leaving the majority of the areas of blood vessels exposed to other neural cells or extracellular matrix (Figures 4(a) and 4(b)). In the rat brain, CD31, a marker for endothelial cells, was used to demonstrate blood vessels. Stripes or patches of CD31 positive signals were observed on endothelial cells with some areas void of CD31 signals (arrows). GFAP-positive astrocytes had relatively ill-defined cell bodies, like what was seen in the human brain. Long processes of astrocytes wrapped blood vessels (arrowheads). Interestingly, it seemed that one astrocyte only wrapped one segment of the blood vessel, and a few of them wrapped multiple segments of the blood vessel (Figures 5(a) and 5(b)). CD146 is a specific marker for pericytes in the brain. Double immunofluorescence staining for both GFAP and CD146 revealed that astrocytes were present in the entire hippocampus without preference, whereas CD146 positive pericytes were primarily present in the CA1 region but not the dentate gyrus at the level of the rostral midbrain. Overlap between the green astrocytes and red pericytes was observed, especially in the ventral portion of the CA1 region (Figures 6(a) and 6(b)), leaving the majority of pericytes being exposed to the extracellular matrix.

## 4. Discussion

The present study examined the 3D structure of the human BBB using the CUBIC technique. It was found that astrocyte end feet did not entirely wrap blood vessels but left a space between two end feet. Glut1 was not evenly distributed in the endothelial cells of blood vessels leaving spots of areas without Glut1. Similarly, Collagen IV staining showed negative spots or stripes along blood vessels. Human BBB showed similar structures to those of the rat brain, which suggests that BBB might be a conserved structure during the evolution. These striking findings are summarized in Table 1.

In contrast to the classical concept that BBB is a structure composed of endothelial cells, the basal membrane, pericytes, and astrocyte end feet that wrap the blood vessels [3], we found that there were spaces between astrocyte end feet. In textbooks and many research articles, a diagram was drawn to show all components of BBB, which leaves an illusion that each part of BBB has the same structure or components. Using GFAP and claudin 5 antibodies, it has been shown that the neonatal brain has astrocyte end feet covering nearly the entire blood vessel, though astrocyte processes are only intermittently present in the space next to the blood vessel [10], which is consistent with what we found in our study. Double staining with CD31 and GFAP antibodies showed incomplete coverage of microvessels by astrocyte end feet. Similarly, double staining with CD146 and GFAP antibodies showed only a small area of overlap between astrocytes and pericytes.

In the present study, astrocytes only wrapped a small portion of blood vessels as shown by GFAP and CD31 or CD146 staining, which is different from the statement of a review that summarized advances in BBB research and reported that astrocyte end feet nearly covered the entire surface of CNS microvessels [11]. This discrepancy might be due to the difference between humans and animals, or between different ages of studied species. Another explanation might be due to the limited effectiveness of the GFAP antibody to stain all astrocytes in the brain. Markers that show all astrocytes will be a better choice to demonstrate the real structure of BBB. In a study focusing on the mapping of blood vessels in the hippocampus and visual cortex, it was found that the hippocampus did have a lower density of capillaries than the visual cortex, which might be related to the decreased blood flow to the hippocampus compared to the visual cortex [18]. The absence of astrocyte end feet on pericytes in the hippocampus, especially in the CA1 region, poses an assumption that lack of neurovascular coupling is likely to be the key reason of reduced blood flow to the hippocampus in addition to the low density of capillaries.

The areas of blood vessels void of astrocyte end feet might be related to transgression of leukocytes into the brain parenchyma. Under the normal condition, this structure is able to stop leukocytes from entering the parenchyma [19–21]. But under ischemia or other conditions, these cells might be able to transgress into the brain parenchyma through these weak points [22, 23], especially in areas that lack Collagen IV in the basal membrane [24]. The other possibility is that these Collagen IV or GFAP negative areas might be where the pericytes are as they are in direct contact with endothelial cells through a peg-and-socket pattern [25].

BBB plays important roles in maintaining neurological functions. It is not only the key structure that impedes the entry of toxic substance from the blood to the brain but also regulates the blood flow of the microcirculation through dilating or constricting the capillaries [26, 27], which is a focus of emerging studies in the past decade. A study by Nortley et al. [28] showed that A*β* elicited pericyte constriction in human brain tissues. In ischemic stroke, ischemia-induced pericyte constriction may persist even after arteries were recanalized [8]. This persistent constriction results in microcirculation insufficiency. In addition, long-lasting ischemia led to pericyte death and entrapment of neutrophils and red blood cells in the microcirculation, aggravating ischemia of the local brain tissue [29–31]. Studies have attempted to clear these neutrophils and red blood cells with anti-Ly6G and observed improvement in neurological functions [31, 32]. So far, little evidence is present from clinical trials on this antibody for ischemic stroke.

Apart from migration of leukocytes into the brain parenchyma, pericytes themselves are able to transform to other types of neural cells, such as microglia. In ischemic stroke, double labelling with both pericyte and microglia markers has confirmed this phenomenon, indicating that pericytes are potent progenitor cells and involved in immune response [33–36].

It has been shown that angiogenesis is also a key step in restoring microcirculation and neurological functions [37]. Pericytes interact with endothelial cells mainly through the TGF-*β*/TGF*β*R2 signalling pathway [38]. Endothelial cells proliferate to form the vessel tubes, and pericytes limit the expansion of endothelial cells and form the tight connection with them to stabilize the vessels and to minimize their permeability [39, 40].

In summary, our study has shown the novel structure of the BBB in 3D. This may have a profound impact in the field of neuroscience. First of all, it provides a good avenue to investigate the BBB structural changes in diverse medical conditions by simultaneously staining the components of the BBB in 3D. The subsequent structural analysis will be more accurate than 2D image analysis. This also applies to studies on changes in angiogenesis during the pathogenesis of various medical disorders. In neurological disorders, such as Alzheimer's disease, the 3D images may reflect more precisely the injury to microvessels and the altered distribution of substances like glucose transporters. This might lead to further insights into the pathogenic mechanisms of this disease. Secondly, the application of tissue clearing and 3D imaging can be extended to other fields to reveal precise spatial relationships of a variety of cells. For example, double staining with tumor markers and immune cell markers will reveal the spatial relationship between these cells and the detailed tumor microenvironment. With fluorophore-conjugated drugs infused into the disease models, it may reveal the spatial relationship between drugs and blood vessels or tumor cells. Therefore, the techniques that we used in the present study and the novel findings from this study will serve as a foundation for future medical research.

The present study also has its own limitations. Pericyte staining with NG2 or PDGFR*β*1 antibody did not work for tissue clearing and staining. In future studies, other markers will be tested, or a transgenic mouse line PDGFR*β*1-EGFP or mCherry will be used to demonstrate the spatial relationship of pericytes with other vascular markers or neural cells to show the entire structure of BBB.

## 5. Conclusions

The present study demonstrated in 3D that Collagen IV, CD31, and Glut1 are unevenly distributed along the blood vessels with some areas void of these signals. Astrocyte end feet do not completely wrap all areas of blood vessels leaving large gaps between their end feet. There are more CD146 positive pericytes in the CA1 region of the hippocampus than in the dentate gyrus, and astrocytes tend to wrap blood vessels in the ventral portion of the CA1 region. Tissue clearing and 3D imaging are promising techniques to answer more questions about the real structure of biological specimens.

## Figures and Tables

**Table 1:** Relatively novel findings from the present study.

**Antibody**	**Interesting findings**
Collagen IV	Unevenly distributed along blood vessels, leaving large areas void of signals
Glut1	Unevenly distributed along blood vessels, leaving large areas void of signals
CD31	Unevenly distributed along blood vessels, leaving large areas void of signals
GFAP	Astrocytes do not completely wrap CD31-positive endothelial cells or CD146-positive pericytes, with the majority of the vessels void of astrocyte end feet; astrocytes seem to wrap one segment of blood vessels rather than wrapping multiple segments of blood vessels using different processes
CD146	More CD146-positive pericytes in the CA1 region of the hippocampus than in the dentate gyrus and astrocytes tend to wrap blood vessels in the ventral portion of the CA1 region
Iba1	In contrast to astrocytes, microglial cells show clear cell bodies

## Data Availability

The data are available from the corresponding author upon request.
